# Increase in wasteosomes (corpora amylacea) in frontotemporal lobar degeneration with specific detection of tau, TDP-43 and FUS pathology

**DOI:** 10.1186/s40478-024-01812-0

**Published:** 2024-06-15

**Authors:** Raquel Alsina, Marta Riba, Agnès Pérez-Millan, Sergi Borrego-Écija, Iban Aldecoa, Clara Romera, Mircea Balasa, Anna Antonell, Albert Lladó, Yaroslau Compta, Jaume del Valle, Raquel Sánchez-Valle, Carme Pelegrí, Laura Molina-Porcel, Jordi Vilaplana

**Affiliations:** 1 Secció de Fisiologia, Departament de Bioquímica i Fisiologia, Facultat de Farmàcia i Ciències de l’Alimentació, Universitat de Barcelona, Avda. Joan XXIII 27-31, 08028 Barcelona, Spain; 2 Institut de Neurociències (UBNeuro), Universitat de Barcelona, Barcelona, Spain; 3 Centros de Biomedicina en Red de Enfermedades Neurodegenerativas (CIBERNED), Madrid, Spain; 4 Alzheimer’s Disease and Other Cognitive Disorders Unit, Neurology Service, Hospital Clínic de Barcelona, Fundació de Recerca Clínic Barcelona-Institut d’Investigacions Biomèdiques August Pi i Sunyer (FRCB-IDIBAPS), Universitat de Barcelona, Barcelona, Spain; 5 Neurological Tissue Bank of the Biobanc-Hospital Clínic-FRCB-IDIBAPS, Barcelona, Spain; 6 Department of Pathology, Biomedical Diagnostic Center (CBD), Hospital Clínic de Barcelona, FRCB-IDIBAPS, Universitat de Barcelona, Barcelona, Spain; 7 Parkinson’s Disease and Movement Disorders Unit, Neurology Service, Hospital Clínic de Barcelona, FRCB-IDIBAPS, European Reference Network On Rare Neurological Diseases (ERN-RND), Agència de Gestió d’Ajuts Universitaris i de Recerca (AGAUR), Barcelona, Spain

**Keywords:** Frontotemporal lobar degeneration (FTLD), Wasteosomes, Corpora amylacea, Tau, TDP-43, FUS

## Abstract

Wasteosomes (or corpora amylacea) are polyglucosan bodies that appear in the human brain with aging and in some neurodegenerative diseases, and have been suggested to have a potential role in a nervous system cleaning mechanism. Despite previous studies in several neurodegenerative disorders, their status in frontotemporal lobar degeneration (FTLD) remains unexplored. Our study aims to characterize wasteosomes in the three primary FTLD proteinopathies, assessing frequency, distribution, protein detection, and association with aging or disease duration. Wasteosome scores were obtained in various brain regions from 124 post-mortem diagnosed sporadic FTLD patients, including 75 participants with tau (FTLD-tau), 42 with TAR DNA-binding protein 43 (FTLD-TDP), and 7 with Fused in Sarcoma (FTLD-FUS) proteinopathies, along with 29 control subjects. The wasteosome amount in each brain region for the different FLTD patients was assessed with a permutation test with age at death and sex as covariables, and multiple regressions explored associations with age at death and disease duration. Double immunofluorescence studies examined altered proteins linked to FTLD in wasteosomes. FTLD patients showed a higher accumulation of wasteosomes than control subjects, especially those with FTLD-FUS. Unlike FTLD-TDP and control subjects, wasteosome accumulation did not increase with age in FTLD-tau and FTLD-FUS. Cases with shorter disease duration in FTLD-tau and FTLD-FUS seemed to exhibit higher wasteosome quantities, whereas FTLD-TDP appeared to show an increase with disease progression. Immunofluorescence studies revealed the presence of tau and phosphorylated-TDP-43 in the periphery of isolated wasteosomes in some patients with FTLD-tau and FTLD-TDP, respectively. Central inclusions of FUS were observed in a higher number of wasteosomes in FTLD-FUS patients. These findings suggest a role of wasteosomes in FTLD, especially in the more aggressive forms of FLTD-FUS. Detecting these proteins, particularly FUS, in wasteosomes from cerebrospinal fluid could be a potential biomarker for FTLD.

**Supplementary Information:**

The online version contains supplementary material available at 10.1186/s40478-024-01812-0.

## Introduction

*Corpora amylacea* are polyglucosan aggregates that appear in the human brain during aging [6, 8] and accumulate in large numbers in specific areas of the brain in some neurodegenerative diseases, such as Alzheimer’s disease (AD) [3, 10, 16, 54, 58], Parkinson’s disease [40, 58], Huntington’s disease [3], amyotrophic lateral sclerosis [40] or multiple sclerosis [53, 54], and also in other brain disorders, as temporal lobe epilepsy [27, 30, 36], vascular encephalopathy [26] and sleep apnoea [59]. Despite their discovery in the nineteenth century, corpora amylacea have been considered irrelevant for a long time [7, 45]. However, in recent decades, they have gained attention for their potential implications in clinical practice.

*Corpora amylacea* are spherical structures ranging from 2 to 20 µm in diameter [8] situated in the cytoplasm of astrocytes [1, 28, 41, 51]. Immunolabeling challenges have led to debates on their composition [2, 47]. It is currently known that they are 68.4% hexoses and 8.1% proteins, while 23.5% remain unidentified [50]. In terms of the carbohydrate portion, *corpora amylacea* consist of polymers of mainly glucose and contain some neo-epitopes of carbohydrate nature [43]. Neo-epitopes are epitopes formed de novo in physiological or pathophysiological conditions, usually related to the elimination of waste substances and homeostasis and are recognized by natural IgMs [21]. Natural antibodies play a role in immune homeostasis by binding to dead, dying, and senescent cells, detecting glycans acting as “eat-me” signals [4, 19, 23]. Regarding proteins, *corpora amylacea* contain glycogen synthase and p62, primarily located in the peripheral region of the polyglucosan structure. Additionally, ubiquitin is found in the peripheral area and can also be concentrated in a central core [2, 9, 10]. This unique composition and content distribution suggest that *corpora amylacea* gather ubiquitinated substances, captured by the surrounding p62 and then trapped in the polyglucosan scaffold constructed by glycogen synthase [2]. Furthermore, degenerated mitochondria, membranous vesicles, and products derived from neurons, glia, blood, and even bacteria or fungus have been described inside *corpora amylacea* [1, 8, 35, 39, 40, 48, 51].

*Corpora amylacea* can be found in almost all regions of the central nervous system, with notable accumulation in perivascular, periventricular and subpial regions, which are close or related to the cerebrospinal fluid (CSF) [8, 50]. Accordingly, in 1993, some authors suggested the possible extrusion of *corpora amylacea* from the brain parenchyma to the CSF as part of a complex brain-cleaning mechanism [51]. This hypothesis was proved years later by finding *corpora amylacea* in post-mortem intraventricular CSF [42]. Moreover, as CSF drains through the recently rediscovered meningeal lymphatic capillaries towards the cervical lymph nodes [31], *corpora amylacea* could reach the cervical lymph nodes [42]. C*orpora amylacea* can be phagocytized in vitro by macrophages, and they can also interact with macrophages placed in the cervical nodes or in the brain interfaces [42, 44]. All this evidence reinforces the notion that *corpora amylacea* could be part of a complex brain-cleaning mechanism. In line with this function, and to avoid any confusion between fibrillary amyloid proteins and the term”amyloid” used for starch-like structures, we have proposed to rename *corpora amylacea* as “wasteosomes” (indicating a body containing waste) [46]. Henceforth, we will refer to them as wasteosomes.

Misfolding and aggregation of specific proteins such as amyloid-beta and hyperphosphorylated tau or α-synuclein are hallmark events in several neurodegenerative diseases such as AD or Lewy body disease, respectively. Many studies have attempted to detect these proteins within wasteosomes with controversial results [5, 12, 29, 37, 58], probably due to false staining caused by IgMs [2]. Notably, recent studies have considered the particularities of wasteosome immunolabeling, and have found tau in wasteosomes from AD patients [47, 57], but not in wasteosomes from healthy control patients [47]. These findings suggest that the composition of wasteosomes may vary according to neuropathological findings.

Frontotemporal lobar degeneration (FTLD), the second most prevalent cause of dementia in individuals under 65 years of age [14] constitutes a clinically, genetically, and pathologically heterogeneous spectrum of disorders characterized by degeneration of the frontal and temporal lobes, resulting in a progressive decline in cognition, language, behavior, and motor function. Neuropathologically, FTLD can be categorized into three main subtypes: FTLD related to misfolded tau (FTLD-tau), FTLD associated with TAR DNA-binding protein 43 (FTLD-TDP) and FTLD with Fused in Sarcoma (FTLD-FUS), based on the accumulation of three major proteins (tau, TDP-43 and FUS) in affected neurons and glia. While FTLD-tau and FTLD-TDP are the two most prevalent subtypes, FTLD-FUS accounts for only 5–10% of FTLD patients [20, 32]. Furthermore, FTLD-tau can be subdivided into 4-repeat isoform (4R) tauopathies, such as progressive supranuclear palsy (PSP) or corticobasal degeneration (CBD), or 3-repeat isoform (3R) tauopathies, such as Pick’s disease (PiD) [33].

To date, no study has explored the association between wasteosomes and FTLD, and as mentioned before, since wasteosomes may accumulate different proteins based on the underlying condition and are extruded into the CSF, they could serve as a potential biomarker for FTLD. Therefore, this study aims to examine the presence, accumulation, and localization of wasteosomes in FTLD and determine the presence of proteins related to FTLD within them to verify their potential use as a biomarker.

## Materials and methods

### Study participants

The study included 153 brain tissue donors from the Neurological Tissue Bank (NTB, Biobank-Hospital Clínic-Fundació de Recerca Clínic Barcelona (FRCB)-Institut d’Investigacions Biomèdiques August Pi i Sunyer (IDIBAPS), Barcelona): 124 patients with a confirmed neuropathological diagnosis of FTLD and 29 non-diseased control participants. Genetic FTLD patients were excluded. Briefly, the NTB was established in the late 80s as a tool for the diagnosis and research of neurodegenerative diseases. It has collected neurological tissue from over 2300 subjects (99% European descent) through a brain donation program covering the Catalonia region (7.7 million inhabitants) in Spain. All participants and/or their legal representatives provided written informed consent to obtain the brain tissue samples for research purposes. The Bioethical Committee of the University of Barcelona approved this project in accordance with the Declaration of Helsinki (IRB00003099).

### Neuropathology

The neuropathological examination was performed according to the standardized protocols at the NTB and following international consensus criteria [34]. Briefly, for each donor, one-half of the brain was fixed in a 4% formaldehyde solution for three weeks, and at least 25 brain regions were embedded in paraffin, cut at 5 µm, and stained with hematoxylin and eosin, as well as luxol fast blue in selected brain areas. Immunohistochemistry was performed using antibodies anti-amyloid β, anti-phospho-tau, anti-RD3 and anti-RD4 tau, anti-α-synuclein, anti-ubiquitin, anti-p62, anti α-internexin, anti-FUS, anti-TDP-43, and anti-phosphorylated-TDP-43 (pTDP-43) (see Supplementary Table 1 for detail). The other half of the brain was dissected fresh, and a hippocampal fragment was obtained, which was immersed in a 4% paraformaldehyde solution at 4 °C for 24 h, followed by 48 h in a 30% sucrose solution in phosphate-buffered saline (PBS) at 4 °C. Subsequently, once dried, the hippocampus fragment was frozen at − 20 °C and stored at − 80 °C. Finally, 6 μm sections were obtained in the cryostat to perform immunofluorescence and detect proteins in wasteosomes.

### Wasteosomes score

In evaluating the accumulation of wasteosomes in brain tissue, we examined hematoxylin and eosin-stained paraffin-embedded sections from five brain regions: superior frontal gyrus, hippocampus, lentiform nucleus, calcarine sulcus in the occipital cortex and medial superior temporal. Using an optical microscope, we assessed wasteosome presence in specific regions within the superior frontal gyrus, lentiform nucleus, calcarine sulcus and medial superior temporal, including subpial, periventricular (applicable only in sections from the lentiform nucleus), perivascular, and intraparenchymal area. In the hippocampal region, extensively investigated in other pathologies or aged individuals, we focused on seven specific areas: the dentate gyrus, fimbria, fimbriodentate sulcus, hippocampal sulcus, subiculum, pre-subiculum and periventricular area.

Distinct patterns of wasteosome accumulation were observed in each area. For instance, in the subpial area, wasteosomes tended to arrange in different rows along the tissue border close to the pia mater, primarily concentrating in the depths of sulci. Similarly, the periventricular area exhibited wasteosome accumulation in rows near the ependyma. In the perivascular area, wasteosomes typically organized in rows immediately following the perivascular spaces, although some were found in isolation within the tissue surrounding the vessel, deviating from the row pattern. Conversely, intraparenchymal wasteosomes did not exhibit a specific pattern.

**Fig. 1:**
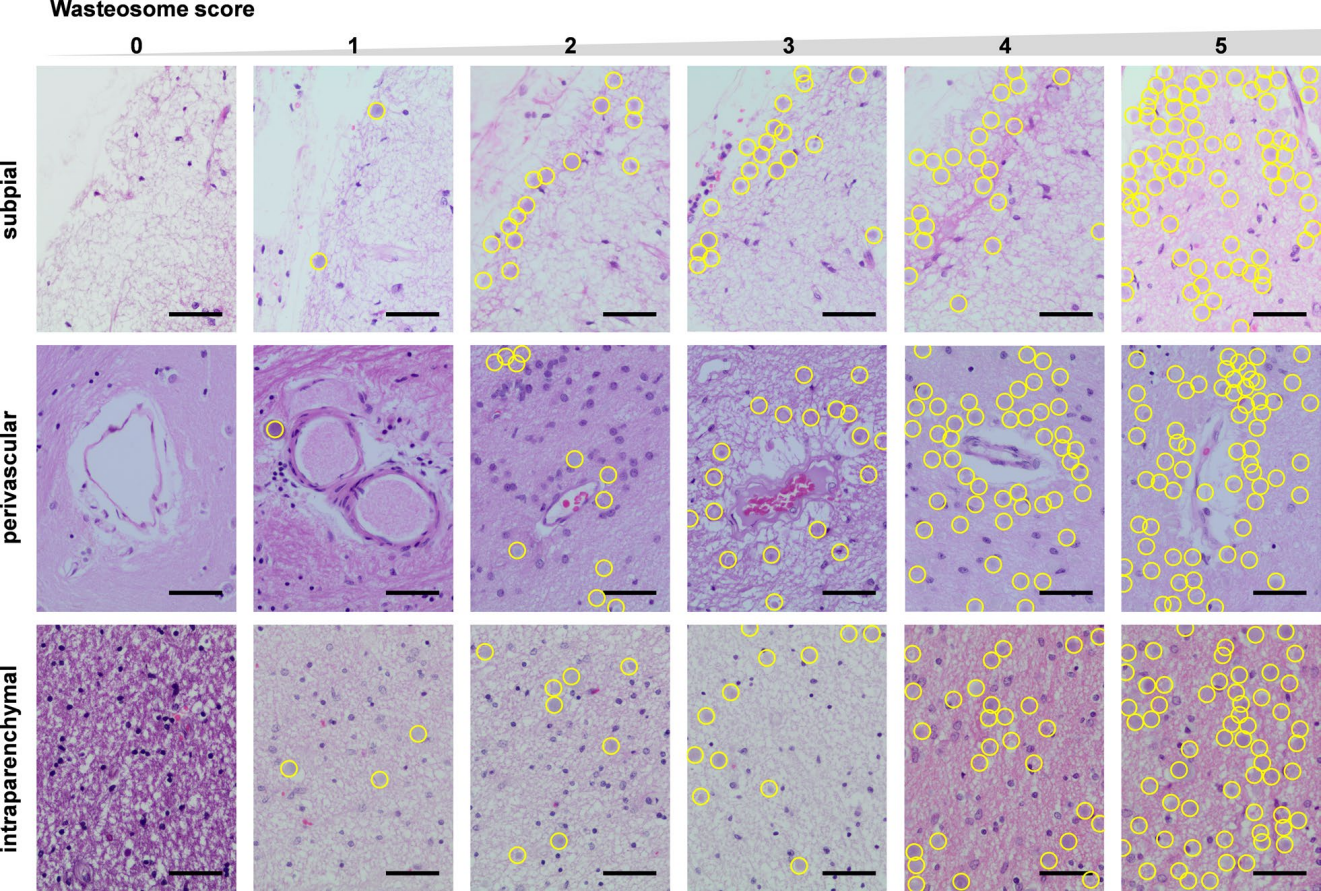
Wasteosome score in the subpial, perivascular, and intraparenchymal areas, shown with representative images from hematoxylin and eosin-stained sections. The first row shows five subpial areas in the temporal lobe region, scoring from 0 to 5. The second row presents five perivascular areas in the lentiform nucleus region, also scoring from 0 to 5. Finally, the third row shows five intraparenchymal areas in the fimbria of the hippocampus, scored from 0 to 5. Each wasteosome is highlighted with a yellow circumference. Scale bar: 50 µm

Based on the different wasteosome accumulation patterns, a specific scale was designed for each area and wasteosomes were manually counted by a single researcher who was blinded to the pathological diagnosis. For instance, in the subpial area, we focused on the depth of sulci, assigning a score of 0 (no wasteosomes) to 5 (≥ 2 rows + high density of wasteosomes surrounding the rows) to each sulcus and then calculated the mean value of all of them. Similarly, to determine the score in the perivascular area, we examined all vessels with a diameter greater than 10 μm in 3 specific fields of view (FOV) at 100 × and scored each vessel from 0 (no wasteosomes) to 5 (≥ 2 rows of wasteosomes + high density of wasteosomes in the tissue close to the vessel), also averaging the scores. However, in the periventricular and intraparenchymal areas, we focused on the FOV at 400 × with the highest accumulation of wasteosomes. In the selected FOV at 400 × in the periventricular area, we applied the same quantification as in the subpial area. In the selected FOV at 400 × in the intraparenchymal area, as there was no specific accumulation pattern, we counted the number of wasteosomes and assigned a value ranging from 0 (no wasteosomes) to 5 (> 60 wasteosomes). Finally, an average of the scores from the different areas and regions per subject was computed, as not all participants had all regions or areas evaluated. Specifically, temporal lobe sections from 57 participants and lentiform nucleus sections from 52 participants were not available. Consequently, the global wasteosome score for each subject ranged from 0 to 5. Representative images exhibiting wasteosome score in the subpial, perivascular and intraparenchymal areas are shown in Fig. 1. Concerning the hippocampal areas, the dentate gyrus and the fimbria were scored as an intraparenchymal area, while the fimbriodentate sulcus, the hippocampal sulcus, the subiculum and the pre-subiculum were scored as a subpial area. See Supplementary Table 2 for more information.

### Statistical analysis

Differences between groups regarding age at death, disease evolution, and post-mortem delay, were evaluated using permutation tests. Differences between groups regarding sex were evaluated using the Fisher test. The *p* values of these results were adjusted for multiple comparisons with the Benjamini & Hochberg correction.

Permutation tests with age at death and sex added as covariables were also used to study the differences in the wasteosome score in the brain tissue. The *p* values of these results were adjusted for multiple comparisons with the Benjamini & Hochberg correction. We compared controls against FTLD patients. Then, we subdivided the FTLD group based on the proteinopathy (FTLD-tau, FTLD-TDP and FTLD-FUS) and all variables were analyzed with the same procedure.

Multiple linear regression adjusted by sex was applied to evaluate the association between the mean of wasteosome score and the age at death or the disease duration.

For all the analyses, statistical significance was set at *p* value < 0.05. Statistical analyses were conducted using R in R-studio version 4.2.1.

### Human brain samples for protein detection in wasteosomes

Post-mortem cryopreserved hippocampal sections (thickness of 6 µm) were obtained from 10 cases of neuropathologically confirmed FTLD patients: 4 cases of FTLD-tau (2 cases of CBD, 1 case of PiD and 1 case of PSP), 3 cases of FTLD-TDP, and 3 cases of FTLD-FUS (demographic and neuropathological data in Supplementary Table 3). Neuropathological examination was performed using the paraffin-embedded sections from half of the brain, as explained in the neuropathology section.

### Immunofluorescence

Hippocampal sections were air dried for 10 min at room temperature. Subsequently, they were placed in a water bath set at 100 °C inside staining dishes containing citrate buffer (pH 6.0) for 7.5 min. The staining dishes were then removed from the water bath and left at room temperature for 20 min. After cooling down, the samples were washed with PBS. Following the heat pre-treatment, hippocampal sections were blocked and permeabilized with 1% bovine serum albumin (Sigma-Aldrich, Madrid, Spain) in PBS (blocking buffer, BB) containing 0.1% Triton X-100 (Sigma-Aldrich) for 20 min. Samples were then washed with PBS and incubated for 21 h at 4 °C with the primary antibodies for double staining: a mouse monoclonal IgG_1_ against tau or a mouse monoclonal IgG_1_ against pTDP-43 or a mouse monoclonal IgG_1_ against FUS/TLS, and a mouse monoclonal IgG_2a_ against p62 (antibody data is compiled in Supplementary Table 1). Sections were then washed and incubated for 1 h at room temperature with the corresponding secondary antibodies: AF555 goat anti-mouse IgG_1_ (1:250; A-21121; Life Technologies; Eugene, OR, USA) and AF488 goat anti-mouse IgG_2a_ (1:250; A-21137; Life Technologies). Nuclei were then stained with the Hoechst stain (2 μg/mL; H-33258; Fluka; Madrid, Spain), and the samples were washed and coverslipped with Fluoromount (Electron Microscopy Sciences; Hatfield, PA, USA). Staining controls were performed by incubating with BB instead of the primary antibody before incubation with the secondary antibody.

### Image acquisition

Images were taken with a fluorescence laser and optical microscope (BX41, Olympus, Hamburg, Germany) and stored in.tiff format. Images were acquired with the 40 × objective, using specific laser and software settings to visualize wasteosomes or protein inclusions. Exposure time was adapted to each staining, and the respective control images were acquired with the same exposure time. Image treatment and analysis were performed with the ImageJ program [52]. Images modified for contrast and brightness to enhance their visualization were processed in the same way as their respective controls.

Additionally, image stacks of the double stained slides were taken with a confocal laser scanning microscope at 63 × (LSM 880, Zeiss, Oberkochen, Germany), and 3D animations were obtained using the ImageJ program [52].

## Results

### Cohort characteristics

The study population comprised 153 brain tissue donors from the NTB: 124 patients with a confirmed neuropathological diagnosis of FTLD and 29 non-diseased control participants. Among the 124 FTLD patients, we included all the available patients in the NTB diagnosed with FTLD-TDP (N = 42 patients) and FTLD-FUS (N = 7 patients). For FTLD-tau, we included 75 patients. Specifically, we included all the available cases of CBD (N = 28 patients) and PiD (N = 14 patients), and we selected the 33 patients with PSP who had the lowest degree of concomitant pathology. The number of participants, age at death, years of disease duration, sex and post-mortem delay are summarized in Table 1. There were no significant differences in age at death or disease evolution. However, significant differences in sex were found between all groups. Controls and FTLD-tau patients showed significant differences in the post-mortem delay.

**Table 1:** Characteristics of the donors

	Controls	FTLD-tau	FTLD-TDP	FTLD-FUS	*p* value adjusted
Number of participants (N)	29	75	42	7	–
Age at death (years, mean ± SD)	73 ± 14.7	73 ± 8.5	73 ± 11.0	59.3 ± 15.0	NS
Disease evolution (years, mean ± SD)	–	8 ± 4.0	7.8 ± 4.7	6.1 ± 3.9	NS
PMD (hours, mean ± SD)	12 ± 6.9	8 ± 4.2	9 ± 4.6	8.6 ± 6.7	0.034^a^
Sex (% female)	65.5	53.3	38.1	28.6	< 2.0e−16^a^
					< 2.0e−16^b^
					1.2e−7^c^
					< 2.0e−16^d^
					3.9e−10^e^
					1.4e−8^f^

Differences between groups regarding age at death, disease evolution, and post-mortem delay, were evaluated using permutation tests. Differences between groups regarding sex were evaluated using the Fisher test. *p* values adjusted by multiple comparisons are only shown for the significant cases. NS: not significant; PMD: post-mortem delay; SD: standard deviation. ^a^Controls vs FTLD-tau; ^b^controls vs FTLD-TDP; ^c^controls vs FTLD-FUS; ^d^FTLD-tau vs FTLD-TDP; ^e^FTLD-tau vs FTLD-FUS; ^f^FTLD-TDP vs FTLD-FUS

### Analysis of the accumulation of wasteosomes

**Fig. 2:**
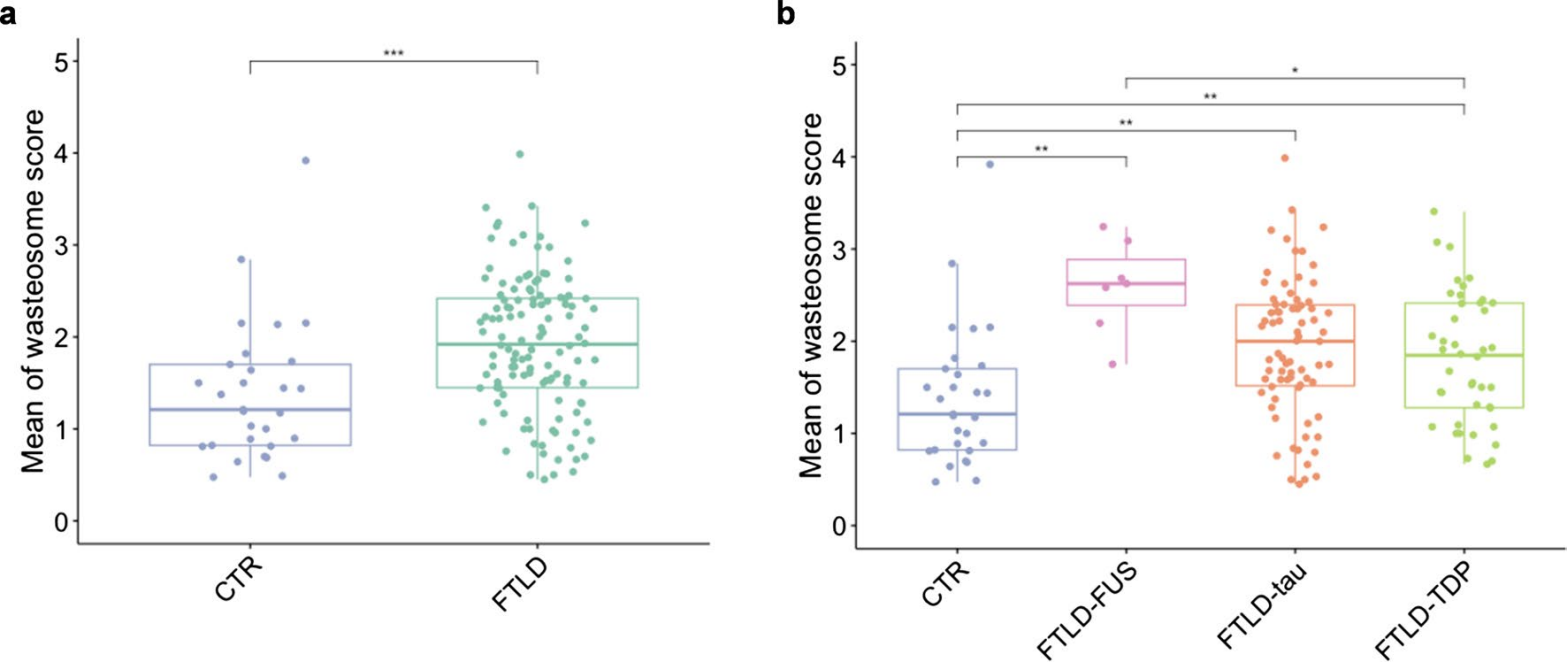
Wasteosome accumulation in FTLD. **a** FTLD showed a higher accumulation of wasteosomes compared to the controls (permutation test, ***adjusted *p* value =  < 0.001). **b** All FTLD subtypes accumulated higher numbers of wasteosomes compared to the control group. Notably, FTLD-FUS showed a greater accumulation compared to FTLD-TDP (permutation test, **adjusted *p* value < 0.01, *adjusted *p* value < 0.05). CTR: control group; FTLD: frontotemporal lobar degeneration

First, we aimed to assess the overall accumulation of wasteosomes throughout the brain in FTLD. As described in the methods section, we examined five regions: superior frontal gyrus, hippocampus, lentiform nucleus, calcarine sulcus and medial superior temporal. In each region, we quantified the presence of wasteosomes in four areas: subpial, periventricular, perivascular, and intraparenchymal. Notably, at the intraparenchymal area, only the fimbria of the hippocampus exhibited wasteosomes. No wasteosomes were detected in any other regions at the intraparenchymal area. We conducted a comparative analysis between FTLD group and control group employing permutation tests. Covariates such as age at death and sex were included and adjustments were made for multiple comparisons. As illustrated in Fig. 2a, the FTLD group demonstrated a significantly higher accumulation of wasteosomes respect to the control group (adjusted *p* value < 0.001). Subsequently, we extended our comparisons to the different subtypes of FTLD (FTLD-tau, FTLD-TDP, and FTLD-FUS) against the control group. Our findings revealed a pronounced increase in the accumulation of wasteosomes across all FTLD subtypes (FTLD-tau, FTLD-TDP, and FTLD-FUS) when compared to the control group (adjusted *p* value < 0.01 in all comparisons). Further, intra-FTLD subtype comparisons indicated that FTLD-FUS showed a more substantial accumulation of wasteosomes compared to FTLD-TDP (adjusted *p* value < 0.05) (Fig. 2b).

Since the different subtypes of FTLD exhibit distinct regional patterns of proteinopathy in the human brain, our aim was to evaluate whether the distribution pattern of wasteosomes corresponds to the most affected regions according to the FTLD subtype. We conducted a regional analysis using the permutation tests, incorporating age at death and sex as covariables and correcting for multiple comparisons. Specifically, we compared the accumulation of wasteosomes across all areas of the five analyzed regions in the human brain. No region showed significant differences except for the hippocampus, particularly, the hippocampal sulcus, the fimbria, and the hippocampal periventricular areas (Table 2). In the hippocampal sulcus, FTLD-tau and FTLD-TDP showed significant differences compared to controls (adjusted *p* value < 0.01 and < 0.05, respectively). Regarding the fimbria, FTLD-FUS exhibited a higher accumulation of wasteosomes compared to FTLD-tau (adjusted *p* value = 0.01) and compared to controls (adjusted *p* value < 0.05). As for the periventricular area of the hippocampus, FTLD-FUS showed a greater accumulation of wasteosomes compared to controls, FTLD-tau and FTLD-TDP (adjusted *p* value < 0.05 in all comparisons).

**Table 2:** Regional analysis of the wasteosome accumulation

	Control	FTLD-tau	FTLD-TDP	FTLD-FUS	*p* value adjusted
Hippocampal sulcus	1.4 ± 1.4	2.6 ± 1.4	2.5 ± 1.4	3.0 ± 0.5	0.0048^a^
					0.012^b^
Fimbria	2.5 ± 1.5	3.0 ± 1.3	2.9 ± 1.4	4.7 ± 0.5	0.010^c^
					0.031^d^
Periventricular area (hippocampus)	0.7 ± 1.1	0.9 ± 1.2	1.0 ± 1.1	2.9 ± 1.5	0.018^c,d,e^

The regional analysis revealed significant differences among FTLD subtypes in the hippocampal sulcus, the fimbria and the periventricular areas of the hippocampus. Data shown as mean ± SD. Differences between groups were calculated using permutation tests with age at death and sex added as covariables. *p* values adjusted for multiple comparisons are only shown for the significant cases. ^a^Controls vs FTLD-tau; ^b^controls vs FTLD-TDP; ^c^FTLD-tau vs FTLD-FUS; ^d^controls vs FTLD-FUS; ^e^FTLD-TDP vs FTLD-FUS

**Fig. 3:**
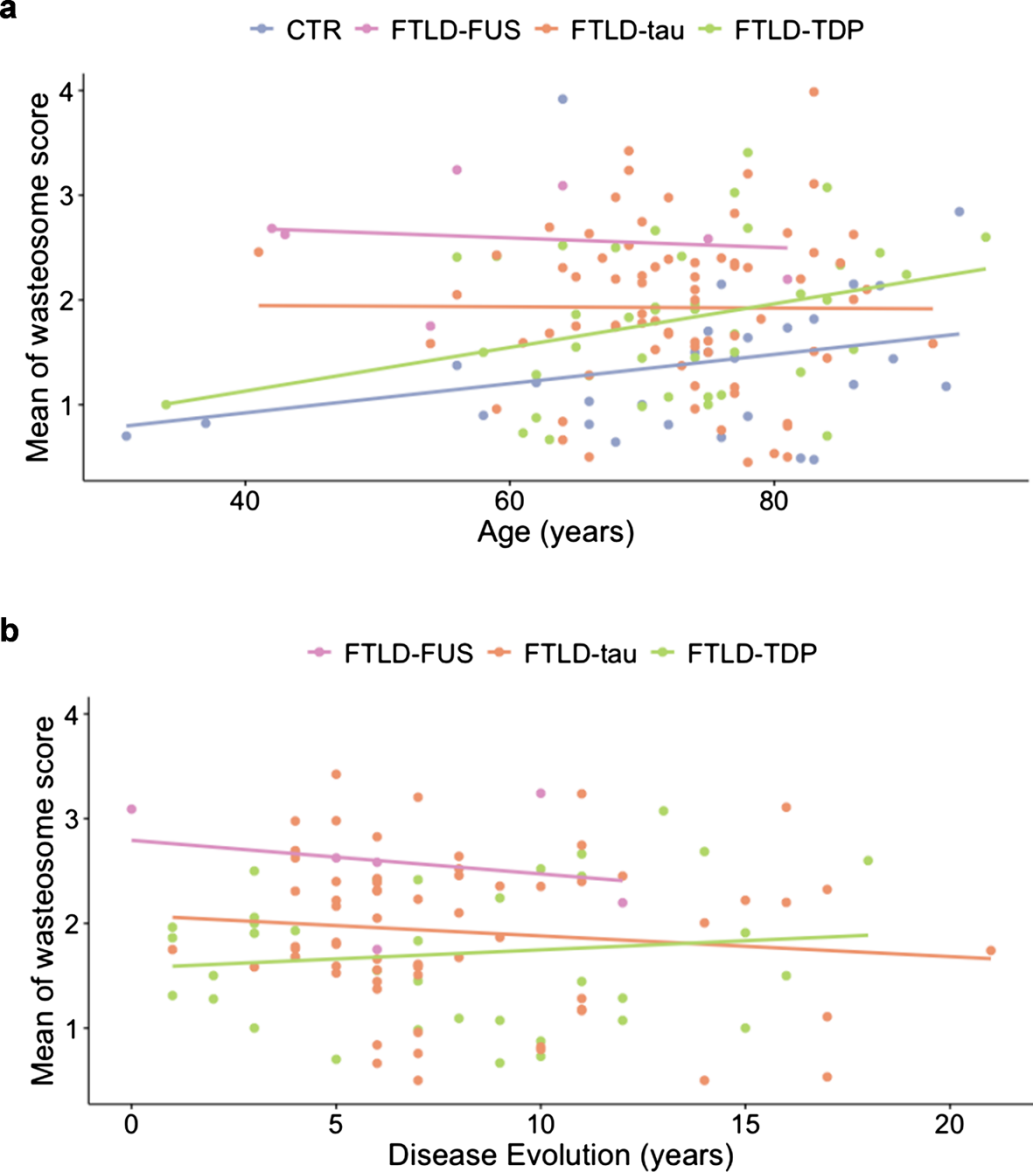
Impact of age at death and disease progression on wasteosome accumulation in FTLD. **a** Impact of age at death in the mean of wasteosome score. Aging increased the number of wasteosomes in controls and FTLD-TDP, but not in FTLD-tau and FTLD-FUS. **b** Impact of disease duration on the quantity of wasteosomes in the different FTLD subtypes. As shown, the accumulation of wasteosomes seemed to increase with disease duration in FTLD-TDP, while it seemed to decrease in FTLD-FUS and FTLD-tau

Finally, we aimed to examine the impact of aging and years of disease duration on the accumulation of wasteosomes in the different subtypes of FTLD and the control group. As described in the literature [8] and depicted in Fig. 3a, wasteosomes tend to increase with normal aging in the control group. Similarly, albeit more prominently, wasteosomes also increased with aging in FTLD-TDP. However, for FTLD-tau and FTLD-FUS, age did not result in substantial changes in wasteosome accumulation. When comparing the progression of wasteosome numbers based on age at death between the control group and FTLD subtypes, significant differences were observed in all comparisons (*p* value < 0.001 in controls vs FTLD-tau, *p* value < 0.05 in controls vs FTLD-TDP, *p* value < 0.0001 in control vs FTLD-FUS) (Table 3). Furthermore, Fig. 3b illustrates the impact of disease duration on the quantity of wasteosomes in the different FTLD subtypes. We observed that, in FTLD-TDP, the accumulation of wasteosomes seemed to increase with disease duration, while in FTLD-FUS and FTLD-tau, it seemed to decrease. Since we lacked a control group for disease progression, and the FTLD-TDP group exhibited a behavior more similar to normal aging, we used it for conducting comparisons and observed significant differences with FTLD-FUS (*p* value < 0.01) (Table 3). No sex differences were observed in either the age at death or the disease progression analysis.

**Table 3:** Association between the accumulation of wasteosomes and age at death or disease duration

	*Β*	SD	*p* value
**Age at death**			
*R*^*2*^* = 0.11*			
Intercept	0.6668	0.4502	0.14
Age at death	0.0099	0.0056	0.081
Controls vs FTLD-tau	0.5471	0.1589	**0.00075**
Controls vs FTLD-TDP	0.4327	0.1771	**0.016**
Controls vs FTLD-FUS	1.3540	0.3157	**3.24e**−**05**
Female vs male	− 0.0170	0.1241	0.89
**Disease duration**			
*R*^*2*^* = 0.05*			
Intercept	1.7656	0.1890	**1.95e−15**
Disease duration	− 0.0053	0.0157	0.73
FTLD-TDP vs FTLD-tau	0.2053	0.161	0.16
FTLD-TDP vs FTLD-FUS	0.8810	0.2873	**0.0028**
Female vs male	− 0.0250	0.1368	0.86

Multiple linear regression evaluated the association between the accumulation of wasteosomes and age at death or disease duration. Multiple linear regression coefficients are shown and significant group differences are highlighted in bold. SD: standard deviation

### Protein detection in wasteosomes from hippocampal tissue

We employed hippocampal sections obtained from 10 FTLD patients: 4 patients of FTLD-tau (2 CBD, 1 PSP, and 1 PiD), 3 patients of FTLD-TDP and 3 patients of FTLD-FUS. For each subject, we performed three double stainings using the anti-p62 antibody, which marks wasteosomes, along with antibodies against tau, pTDP-43, and FUS. This allowed us to investigate the presence of these three proteins in the wasteosomes of all subjects, irrespective of the FTLD subtype.

Consistent with the proteinopathy associated with the different FTLD subtypes, pathological aggregates of tau were exclusively observed in sections from FTLD-tau (PSP, CBD and PiD). Regarding pTDP-43, the positive immunostaining in the hippocampal sections that we examined was confined to sections from FTLD-TDP. Moreover, FUS-positive immunostaining was only observed in sections from FTLD-FUS. Specifically, we identified tufted astrocytes and globose neurofibrillary tangles in PSP, astrocytic plaques in CBD, and characteristic Pick bodies in PiD, exhibiting dual staining with p62 and tau. Similarly, in FTLD-TDP, dystrophic neurites and cytoplasmatic and intranuclear inclusions were observed, stained with anti-pTDP-43 and anti-p62 antibodies. In FTLD-FUS, both intranuclear and cytoplasmatic inclusions were stained with anti-FUS and anti-p62 antibodies. These results substantiate the specificity of the staining performed according to the FTLD subtype. See Supplementary Fig. 1 for details.

**Fig. 4:**
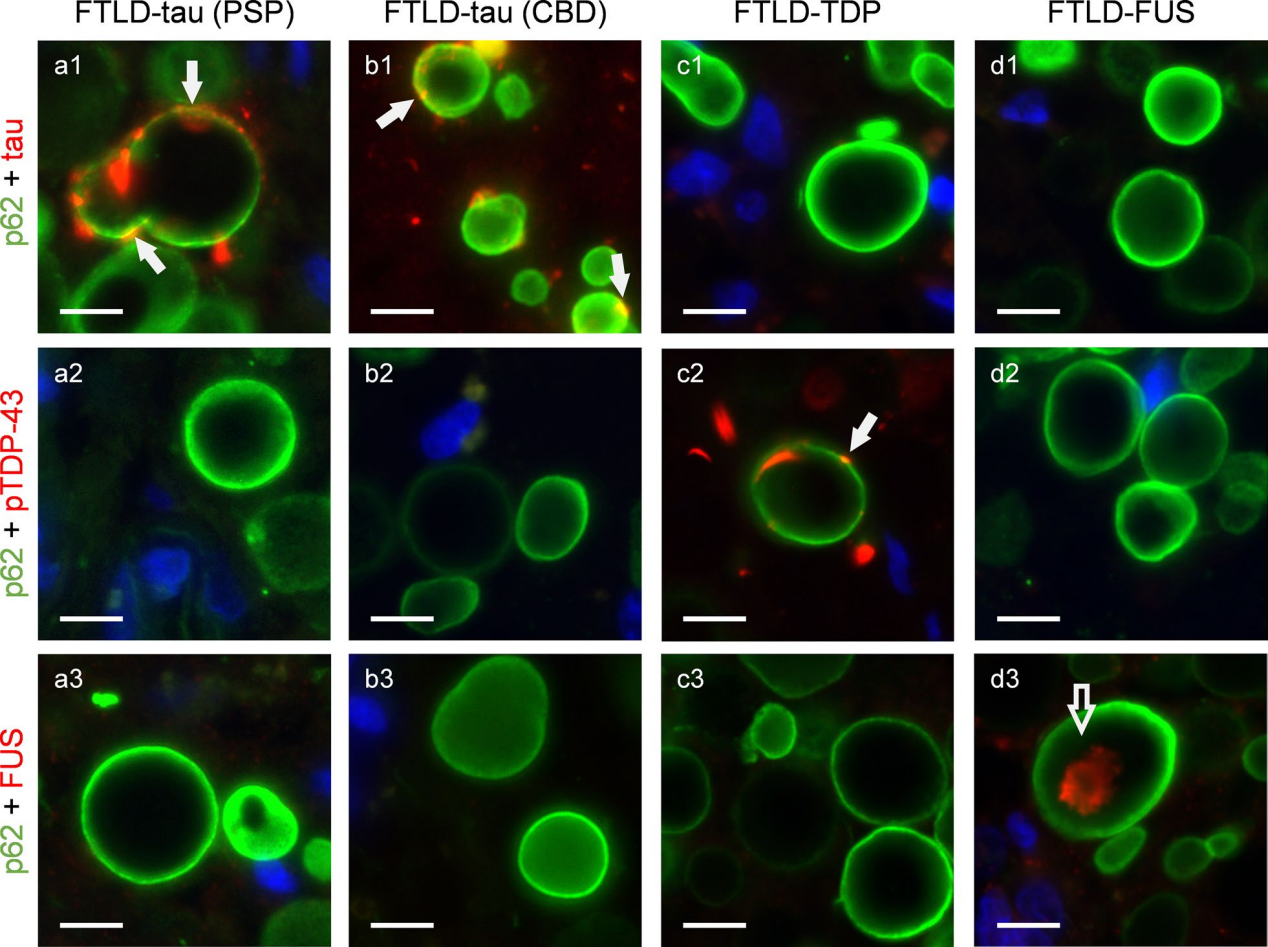
Presence of disease-related proteins in wasteosomes. **a1-a3** Wasteosomes from the hippocampus of a PSP patient stained with anti-p62 (green) and anti-tau (red, **a1**), and not labelled with anti-pTDP43 (red, **a2**) and anti-FUS (red, **a3**). The white arrows in **a1** show tau staining in the periphery of wasteosomes. **b1-b3** Wasteosomes from the hippocampus of a CBD patient stained with anti-p62 (green) and anti-tau (red, **b1**), and not labelled with anti-pTDP43 (red, **b2**) nor anti-FUS (red, **b3**). The white arrows in **b1** show tau staining in the periphery of wasteosomes. **c1-c3** Wasteosomes from the hippocampus of an FTLD-TDP patient stained with anti-p62 (green) and anti-pTDP43 (red, **c2**), and not labelled with anti-tau (red, **c1**) nor anti-FUS (red, **c3**). The white arrow in **c2** shows pTDP-43 staining in the periphery of a wasteosome. **d1-d3** Wasteosomes from the hippocampus of an FTLD-FUS patient stained with anti-p62 (green) and anti-FUS (red, **d3**), and not labelled with anti-tau (red, **d1**) nor anti-pTDP43 (red, **d2**). The empty arrow points out a FUS inclusion in the central area of a wasteosome (red, **d3**). Nuclei are stained with Hoechst (blue). CBD: corticobasal degeneration; FTLD: frontotemporal lobar degeneration; PSP: progressive supranuclear palsy. Scale bar: 10 µm

After verifying the specificity of each immunostaining, we proceeded to investigate the presence of tau, pTDP-43 and FUS proteins within the hippocampal wasteosomes of patients with FTLD. In FTLD-tau we observed colocalization of p62 and tau immunostaining in the periphery of isolated wasteosomes from PSP and CBD patients (Fig. 4a1, 4b1). However, no tau immunostaining was observed in any wasteosomes in PiD. Similarly, wasteosomes from FTLD-TDP and FTLD-FUS patients did not exhibit tau immunostaining (Fig. 4c1 and 4d1, respectively). For pTDP-43 immunostaining, hippocampal sections from non-FTLD-TDP patients (i.e. FTLD-tau and FLTD-FUS) displayed no positive immunostaining (Fig. 4a2, b2, d2), whereas hippocampal sections from FTLD-TDP patients showcased colocalization of p62 and pTDP-43 immunostaining in isolated wasteosomes (Fig. 4c2). Positive tau and pTDP-43 immunostaining in wasteosomes were detected in those wasteosomes proximal to regions abundant in tau and pTDP-43 aggregates. Turning to FUS, hippocampal sections from FTLD-FUS patients revealed FUS positive immunostaining in the central core of a high number of wasteosomes (Fig. 4d3). In this instance, no colocalization between p62 and FUS was observed, as p62 is mainly located in the periphery of wasteosomes. In contrast, we did not observe positive FUS immunostaining in sections from non-FTLD-FUS patients (i.e. FTLD-tau and FTLD-TDP) (Fig. 4a3, b3, c3).

**Fig. 5:**
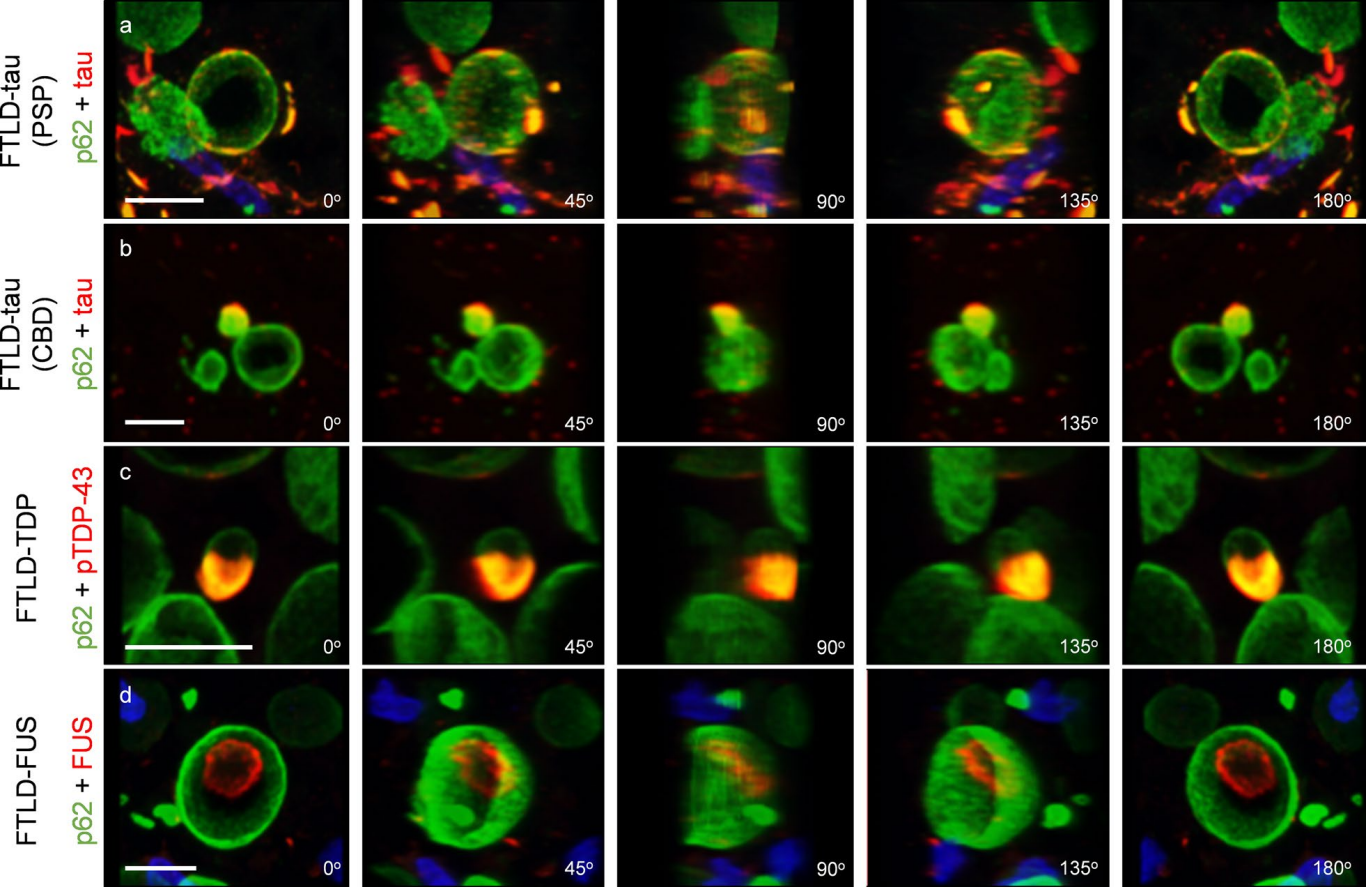
Frames obtained from 3D reconstructions of image stacks. **a** Sequence of images showing 180° rotation of the 3D reconstruction of wasteosomes from the hippocampus of a PSP patient stained with anti-p62 (green) and anti-tau (red) highlighting the peripheral location of tau in the wasteosome structure. **b** Sequence of images showing 180° rotation of the 3D reconstruction of wasteosomes from the hippocampus of a CBD patient stained with anti-p62 (green) and anti-tau (red) revealing the peripheral location of tau in the wasteosome structure. **c** Sequence of images showing 180° rotation of the 3D reconstruction of wasteosomes from the hippocampus of an FTLD-TDP patient stained with anti-p62 (green) and anti-pTDP43 (red) exhibiting the peripheral location of pTDP-43 in the wasteosome structure. **d** Sequence of images showing 180° rotation of the 3D reconstruction of wasteosomes from the hippocampus of an FTLD-FUS patient stained with anti-p62 (green) and anti-FUS (red) demonstrating the location of FUS in the central core of the wasteosomes. Scale bar: 10 µm

The detailed distribution of the disease-related proteins and p62 in wasteosomes is illustrated in Fig. 5. 3D reconstructions, derived from the maximum intensity projection images acquired through confocal microscopy from double-stained hippocampal sections, depict the spatial distribution of these proteins. As can be seen, tau protein and pTDP-43 exhibit colocalization with p62 in the periphery of wasteosomes (Fig. 5a–c and Supplementary Videos 1,2,3). In contrast, FUS does not colocalize within p62 in wasteosomes. Instead, it accumulates in the central core, as corroborated by the 3D reconstructions presented in Supplementary Video 4 and Fig. 5d.

## Discussion

This study highlights that an increase in wasteosomes occurs in FTLD, providing further evidence of their association with neurodegenerative diseases. Wasteosomes, given their composition, structure, and location, may act as a mechanism for entrapping and removing waste substances within the human brain [46]. Other well-studied pathways for waste products degradation are the ubiquitin–proteasome system (UPS) and autophagy-lysosome system. Precisely, through the study of genetic variants of FTLD, it has been suggested that a UPS failure could be involved in the pathophysiology of the disease [38, 49]. In this sense, proteasome dysfunction in FTLD could increase the wasteosomes pathway for eliminating residual substances and lead to a higher accumulation of wasteosomes in the brain parenchyma. Analyzing FLTD subtypes, we observed that FTLD-FUS shows a more substantial accumulation of wasteosomes compared to FTLD-TDP, suggesting differences in their pathophysiological mechanisms.

On the other hand, we observed that wasteosomes tend to increase with aging in the control group, as described in the literature [8]. Similarly, wasteosomes also increased with aging in FTLD-TDP, albeit more pronouncedly than in the control group. In fact, an increase in wasteosome-like granules with aging was described in TDP-43 transgenic mice [55]. On the contrary, in FTLD-tau and FTLD-FUS, age did not show a clear effect. In the context of disease evolution, we observed that the FTLD-TDP group exhibited significant differences with FTLD-FUS. These results would reinforce that the mechanisms of wasteosome accumulation may differ in FTLD-TDP compared to other FTLD subtypes, implying that neurodegeneration in FTLD-FUS could play a more significant role in wasteosome formation than aging itself.

In terms of the regional analysis, we observed differences in wasteosome accumulation in the hippocampal sulcus, the fimbria and the periventricular area, in the hippocampal region. However, we did not observe differences in the accumulation of wasteosomes in other regions, although the various subtypes of FTLD show different distribution of the corresponding proteinopathy [13, 22, 24, 25, 56, 60]. Moreover, when comparing FTLD to controls, we observed that wasteosomes increased in approximately the same regions. Thus, these findings may indicate that FTLD proteinopathy does not directly enhance the production of wasteosomes in the region where the protein aggregates appear but might do so indirectly in other brain regions. In essence, the presence of wasteosomes in a specific region may not necessarily indicate an impairment in that area but could provide information about a dysfunction in the brain on a more global level. Alternatively, it could also be considered that not all regions of the brain have the same capacity to form wasteosomes.

Another relevant finding of this study is the specificity observed in wasteosome immunostaining in FTLD-FUS, FTLD-TDP and FTLD-tau in CBD and PSP. However, the absence of tau protein staining within wasteosomes of PiD patients should be noted. The differences in the tau isoform, 3R tau in PiD, and 4R tau in CBD and PSP, could influence the incorporation of tau protein into wasteosomes. Notably, 4R tau assemble more rapidly than 3R tau and are also preferentially engulfed by astrocytes [15, 18]. Nevertheless, further analysis involving additional cases should be conducted to confirm this result.

Previous studies have reported the presence of proteins such as α-synuclein, amyloid-β, or NeuN in wasteosomes [5, 37, 58]. Additionally, TDP-43 immunoreactivity surrounding wasteosomes was observed in schizophrenia and limbic-predominant age-related TDP-43 encephalopathy patients [11, 17]. However, some of these labelings may be subject to controversy due to the presence of contaminant IgMs in primary antibodies [2]. In this study, we used monoclonal primary antibodies and isotype-specific secondary antibodies to avoid false positive staining caused by IgMs, as described in Augé et al.[2]. Further analysis including other techniques, such as mass spectrometry, would reinforce our results. Recent studies avoiding this false positive staining detected tau in wasteosomes from AD patients [47, 57]. These results, coupled with our findings of tau, pTDP-43, and FUS in wasteosomes from FTLD patients, demonstrate the ability to incorporate major proteinopathies into wasteosomes, supporting their role as waste containers. It should be considered that the mechanisms by which substances are integrated into wasteosomes are still unknown. Whether these substances are entrapped freely or appear in wasteosomes as a result of incorporating organelles into these structures [1, 35], remains to be fully elucidated.

Our results revealed distinct distribution patterns of tau, pTDP-43, and FUS within wasteosomes. Specifically, wasteosomes are known to contain p62 primarily in the peripheral area, while ubiquitin, also present in the periphery, predominantly accumulates in the central core of wasteosomes [2]. Both ubiquitin and p62 play a role in waste removal mechanisms through the UPS and autophagy-lysosome system. Despite tau, pTDP-43 and FUS forming aggregates positive for both p62 and ubiquitin, our observations indicated that tau and pTDP-43 are predominantly located in the periphery of wasteosomes. In contrast, FUS exhibited a distinct distribution, concentrating in the central core of wasteosomes, where ubiquitin accumulates exclusively [2]. These variations raise inquiries into the mechanisms underlying tau, pTDP-43 and FUS aggregation and their integration within wasteosomes. Moreover, given that wasteosomes are released into the CSF, these findings highlight the potential value of utilizing them as a diagnostic tool for neurodegenerative diseases.

In summary, our study demonstrates that wasteosomes accumulate in FTLD, as in other neurodegenerative diseases, with specific labeling depending on the predominant proteinopathy. Detecting these proteins in wasteosomes from the CSF could serve as a biomarker, especially in FTLD-FUS.

### Supplementary Information

Supplementary Material 1.

Supplementary Material 2.

Supplementary Material 3.

Supplementary Material 4.

Supplementary Material 5.

Supplementary Material 6.

## Data Availability

The data presented in this study is available in the article or contained in the supplementary information.
