# Supplementary material for: Increase in wasteosomes (corpora amylacea) in frontotemporal lobar degeneration with specific detection of tau, TDP-43 and FUS pathology
Source: Acta Neuropathol Commun. 2024 Jun 15;12:97. doi: 10.1186/s40478-024-01812-0 (PMC11179228; doi:10.1186/s40478-024-01812-0)
Supplement: Supplementary file 1 — Supplementary Material 1. [file 40478_2024_1812_MOESM1_ESM.pdf]

FTLD	Protein	Age at death (years)	Sex	PMD (hours)	Brain weight (g)	Disease evol. (years)	W_SFG_subpial	W_CS_subpial	W_MST_subpial	W_Lent_subpial	W_Lent_PVA	W_Lent_PVE	W_Hc_DG	W_Hc_FDS	W_Hc_HS	W_Hc_Fi	W_Hc_PVE	W_Hc_SB	W_Hc_PSB
No	No	75	F	20,0			1	2,5					2		0		1	2	2
No	No	78	F	0,0			1	1					2	0	0	1	0	2	1
No	No	77	F	20,0			0	1					2	2	1	2	1	2	2
No	No	37	F	24,0			0	0,75					1		2		0	1	1
No	No	66	M	19,0			0	0,5					0	3	0		0	2	1
No	No	66	M	7,0				0,25					1	2	1	1	0	1	2
No	No	68	F	13,0			0	0,5					0		0		2	2	0
No	No	70	M	16,5			0	1					0	3	1	3	0	1	0
No	No	76	M	17,0				0,5					0	0	1	1	0	1	2
No	No	72	F	6,0			1	0,666666667					0		1		1	1	1
No	No	82	M	2,5			0	0,4					0	2	0	2	0	0	0
No	No	74	F	3,7			0	0,5					0		2		2	3	3
No	No	81	F	23,5			0	0,333333333		2			2	3	1	4	0	3	2
No	No	31	M	17,5	1500		0	0,5		0,5			2	0	0	2	0	1	1
No	No	64	M	10,0	1550		2,333333333	2,6	4,25				4	5	3	5	5	4	4
No	No	56	F	14,0	1280		0,25	1,5	2				0	1	1	1	0	4	3
No	No	58	M	5,0	1390		0	0,333333333	1	0,333333333	3	0	1	2	0	2	0	2	0
No	No	78	M	6,0	1210		1	0,2	0,5	3,333333333			2	1	3	2	0	3	2
No	No	76	M	11,5	1295		0,5	0,8	2,142857143	3,5	2	0	2	3	4	4	1	3	2
No	No	88	F	24,0	1075		0,666666667	0,666666667	1,166666667	0			5	2	3	4	3	3	1
No	No	83	F	7,5	1345		0,333333333	1	1,666666667	1,333333333	1,3	1	3	5	1	5	0	1	2
No	No	93	F	5,5	905		0,5	0	0,6	2,666666667	2,5	0	1	2	1	4	0	1	0
No	No	83	F	7,3	1320			0	0	0,75			1	0	0	1	0	2	0
No	No	86	M	7,4	1365		0,833333333	0,75	0,5	1,5	0,384615385	0	5	5	5	4	1	2	2
No	No	94	F	15,8	1245		2,333333333	2,833333333	3,272727273	2,25	4,272727273	2	1	4	3	3	1	4	4
No	No	89	F	8,8	1040		0,125	0,4	0,833333333	3	1,214285714	0	1	2	2	4	1	2	0
No	No	86	F	8,4	1180		0,714285714	0,714285714	1,4	3	1,666666667	0	1	2	1	1	1	1	1
No	No	62	M	18,1	1550		0	0,4	0,333333333	2	5	0	3	1	0	0	1	3	0
No	No	75	F	17,3	1215		0,333333333	0,166666667	2,666666667	4,25	2		1	3	3	2	0	1	1
Yes	FUS	42	M	0,0		4	2	0,333333333		4,5			4	2	2	5	3	2	2
Yes	FUS	43	M	4,0	960	5	0	1		3	2,5	0	1	5	4	5	1	4	5
Yes	FUS	75	F	8,5		6	2	3,333333333		1,333333333			2		4		3	3	2
Yes	FUS	64	M	18,8	1390	0	0,75	0,75	4,5	5			3	5	3	5	3	2	2
Yes	FUS	81	M	6,5	1190	12	0	0,666666667	1,5	3			3	3	2	4	1	4	2
Yes	FUS	56	M	16,4	1225	10	0,285714286	1,142857143	0,888888889	5	4,833333333	1	2	5	5	5	4	5	3
Yes	FUS	54	F	5,8	905	6	0	0,428571429	2,333333333	1	2	1	0	2	1	4	5	2	2
Yes	TDP	56	M	8,0			0	0,5		4		0	0	5	3	5	3	4	2
Yes	TDP	84	F	6,0	985	13	1	2,666666667		4			3	5	4		1	4	3
Yes	TDP	34	M	7,0	1280	3	0	0	2				0		2		1	2	1
Yes	TDP	76	M	5,0	1200	8	0	0,2	0,5	2,5	2	0	0	2	2	3	0	1	1
Yes	TDP	71	M	5,0	1200	11	1	1,2	2,75	5	3	0		4	4	4	1	2	4
Yes	TDP	64	M	10,5	930	10	0,5	2,2	1,545454545		1	0	5	3	4	4	3	3	3
Yes	TDP	71	M	4,0	1220	3	0,5	0,666666667	0,6	3	5	2	4	2	0	2	0	3	2
Yes	TDP	90	F	6,0	810	9	1	1,75	2,25	3,666666667			2	2	4	4	1	2	1
Yes	TDP	77	M	6,3	1195	2	0,444444444	0,5	0,571428571	2	5	0	1	1	2	1	0	3	3
Yes	TDP	82	M	15,8	1290	1	0,2	0,5	0,857142857	1,333333333	2,833333333		1	3	2	1	1	1	1
Yes	TDP	74	F	15,0	1050	7	1	1		4,5	4	0	1		0		0	2	1
Yes	TDP	77	F	6,4	900	4	1	1,4		2			3		3		0	2	1
Yes	TDP	68	F	7,3	1040	3	1	2		5	5	0	2		4		1	2	3
Yes	TDP	65	M	13,0	1210	6	1	0,5		3			0	3	2	3	0	2	1
Yes	TDP	88	F	6,5		11	0	1		2,5			3	4	3	4	1	4	2
Yes	TDP	66	M	5,3	1205	2	0	0	1	3,5			0		1		2	3	1
Yes	TDP	82	F	3,7	945	3	3		0,8		4,428571429	0							
Yes	TDP	84	M	19,3	1055	5	0,166666667	0,2	0,125	0,5	2	0	0	1	1	1	0	1	1
Yes	TDP	69	F	11,8		7					3,5	0	0	1	1	1	2	4	4
Yes	TDP	65	F	4,4	975	1	0,857142857	1,333333333	2,428571429	3	3	0	0	4		3	1		
Yes	TDP	96	F	6,8	1025	18	3	2,363636364	3,875	3,666666667	0,692307692	1	2	4	4	4	0		
Yes	TDP	71	M	7,0		4	1	0,666666667		4,5	3	0	1	3	4	1	0	3	2
Yes	TDP	59	M	8,0	1120	7	0,5	0		2,5	5	2	4	2	2	3	1	3	4
Yes	TDP	84	F	16,0	1290	3	0	2		4			2		1		4	2	1
Yes	TDP	75	M	6,0	1190	15	0	0,5	0	0,5			1	1	3	1	3	1	0
Yes	TDP	86	M	4,0	1105		1	0,375	0,545454545	1,75	1,2	1	2	5	2	4	0	1	0
Yes	TDP	75	M	17,1	1150	9	0,25	0	0,2	2,5	3	0	1	2	0	3	0	1	1
Yes	TDP	61	M	7,8	1010	10	0,5	0		1,333333333			2		0		0	1	1
Yes	TDP	74	F	5,3	1255	1	2	0,666666667					1	2	3	3	2	3	1
Yes	TDP	73	M	5,0		6	0	0,333333333					5	5	3		2	2	2
Yes	TDP	63	M	9,5	1200	9		0					0		1		2	1	0
Yes	TDP	78	M	7,0			0	1,666666667					5	4	5	5	3	4	3
Yes	TDP	85	F	14,7				0,666666667					3	3	3	3	3	2	1
Yes	TDP	58	M	4,0	1280	16	2	0,5					2		3		1	1	1
Yes	TDP	62	M	12,2		12	0	1					1		1		2	2	2
Yes	TDP	70	F	11,0		11	0	1					1	1	2	4	1	2	1
Yes	TDP	78	M	7,3	1165	14	1	0,666666667	1,5	5	2,75	0	5	2	5	5	0	4	3
Yes	TDP	62	F	6,0	1265	10	0	0,25	0,5				0	1	2	1	0	2	2
Yes	TDP	70	M	19,0	1230	7	0,333333333	0,666666667	0,333333333	0,5			1		2	3	0	2	0
Yes	TDP	77	F	16,0	1155		1,25	1	3				1	4	5	5	2	5	3
Yes	TDP	74	M	15,0	1085	15	0,5	0,333333333	0,333333333	0,666666667	4	0	3	2	4	3	0	4	3
Yes	TDP	72	M	7,0	955	12	0	0,125	0,2	3	2,5	0	1	1	1	1	0	2	1
Yes	Tau	86	F	20,0	950	4		1					3	4	4	4	0	3	2
Yes	Tau	72	M	0,0	1200	6	0	2,5					4	5	3	3	0	2	2
Yes	Tau	76	F	6,8	940	11	0	2,6					3	4	4	2	0	3	3
Yes	Tau	70	F	7,0	1020	4	1	0					0	4	3	3	0	3	2
Yes	Tau	68	M	7,0	1280	4	1,333333333	1			5	0	0	2	2	1	1	3	3
Yes	Tau	72	F	10,0	1010	8	0	3,4		4			1		3		1	1	0
Yes	Tau	70	M	15,0	1500	9	1	0		4	0,666666667	0	1		3		5	2	2
Yes	Tau	74	F	4,0	970		0,5	0,5		3			1	3	3	2	3	2	2
Yes	Tau	87	F	8,5	1010	8	1	1		3			3	4	4	1	0	3	1
Yes	Tau	63	F	7,3		4	3	3,25					2	4	2	3	1	3	3
Yes	Tau	74	M	14,5	1065	9	1	1,2					2	4	3	4	1	2	3
Yes	Tau	65	M	16,0	1220	1	0	0,25					1	5	2	4	0		
Yes	Tau	77	F	18,1	1015		0	0,666666667		3,5			1	4	4		0	4	4
Yes	Tau	63	F	8,2	1125	4	1	0,5	1	3			0	3	3	2	0	3	2
Yes	Tau	72	M	7,0	1065		0	1,285714286	1,333333333	2,666666667	3	0	3	5	2		0	1	1
Yes	Tau	66	M	5,0	1250		0,5	1,5	3,6		5	1	5	4	3	4	0	2	2
Yes	Tau	64	M	7,5	1315	4	2	0,666666667	1,666666667	1	3,666666667	2	4	2	2	5	2	2	2
Yes	Tau	71	F	4,8	1145	5	1	0,666666667	2,333333333				2	3	1	4	0	2	2
Yes	Tau	83	M	7,0	1180	7	0,25	1	0,833333333				2	2	1	3	1	2	2
Yes	Tau	74	M	9,0	1195	5	1	1	1,75	2,666666667	4	0	2	4		4	0	4	

Yes	Tau	59	M	7,0	1170	7	0	0,25	1,333333333				0	1	3	1	1	1	1
Yes	Tau	70	F	15,5	110	5	0,666666667	0,5	2,8	4		0	4	3	4	3	1	2	1
Yes	Tau	83	F	16,0	830	16	1	3,75	2,666666667	4	4	0	3	5	4	5	1	3	4
Yes	Tau	78	F	5,5	1340	6	1,8	2,6	4	4			3	3	2	3	0	1	1
Yes	Tau	74	F	8,3	1100	7	0,666666667	1	2	2,666666667	3,25	0	1		1		1	2	3
Yes	Tau	64	M	5,0	1090	6	0	0,8	0,5	1,333333333	2,4	0							
Yes	Tau	81	F	6,7	850	10	0	0	0	1,333333333	0,3125	0	1	2	0	3	0	2	1
Yes	Tau	77	M	6,7		6	0,428571429	1	1,875	3	0,6		2	2	2	3	1	2	1
Yes	Tau	59	M	4,0	1450	6	0	1		3			4	4		4	1		
Yes	Tau	68	F	12,8	1010	21	0	1,666666667					0	2	2	1	1	4	4
Yes	Tau	78	F	5,8	1080	7	2,5	1,75		4		0	3	5	4	5	4	4	2
Yes	Tau	74	M	7,0	1040		0	0		1	2,6	0	1		3		0	1	1
Yes	Tau	54	F	5,1	970	7	0	0		4	4	0	2	3	1	3	1	1	0
Yes	Tau	78	M	2,5	1035		0	0	0,5				0	0	0	1	0	1	2
Yes	Tau	67	M	5,0	1345	5	2	1		3			5	3	3	4	0	2	1
Yes	Tau	83	F	14,4	1035		1	1,857142857	3	5			5	5	5	4	5	5	4
Yes	Tau	81	F	7,5	980	10	0,5	1	0,25	0,5	2,5	0	1		1		0	1	1
Yes	Tau	81	F	5,0	1090	7	0	0	0				1	0	0	0	0	2	2
Yes	Tau	61	F	7,0	1280	5	0	0	1	3,5			3	4	2	1	0	2	1
Yes	Tau	84	F	7,1	1055	6	0	0,5	1,5				2	2	1		1	3	2
Yes	Tau	92	F	16,0	1050	3	1	2,25	1,5										
Yes	Tau	77	F	6,5	970	17	0,4	0	1,25								0	2	3
Yes	Tau	86	F	5,0	965	14	1,5	2,833333333	2,714285714				0	3	3	4	0	2	1
Yes	Tau	77	M	5,6	1010	11	0	0,666666667	0		4								
Yes	Tau	83	M	7,3	1210	12	0,666666667	1,8	2,5	2			2	3	3	5	2	3	2
Yes	Tau	77	M	7,0	1200	6	1	1,666666667	2,6				3	4	4	3	1	4	4
Yes	Tau	64	F	12,0	1380	6	0,25	0,666666667	0,714285714				1	1	1	2	0	0	0
Yes	Tau	80	F	3,5	1260	17	0	0	1,333333333				1	1	0	1	0	1	0
Yes	Tau	79	F	13,5	1295	5	1	1,5	1	3,5			1	2	3	2	1	2	2
Yes	Tau	75	F	13,5	1085	7	0,5	0,5	2,666666667	2	3,25	0	1	3	2	2	1	2	1
Yes	Tau	72	M	16,0	1210	4	1,666666667	1,25	2,833333333	4			4	3	4	3	1	4	4
Yes	Tau	74	M	5,8	1265	11	1,666666667	1	1,8	1,5			0	0	2	2	0	2	1
Yes	Tau	85	F	5,5	960	10	1,5	1,166666667	3	2,666666667	4,25	0	5	3	4	3	1	1	1
Yes	Tau	76	F	14,0	1125	7	0	0,333333333	0,5	2,5			1	0	1	1	1	1	0
Yes	Tau	69	F	6,0	1135	8	0	0,333333333	2,4	5			0	4	3	4	2	4	3
Yes	Tau	81	F	4,0	930	8	0	0,571428571	0,75	5	5	2	3	2	5	3	1	5	2
Yes	Tau	69	M	5,3	1140	5	0,2	2,5	2,142857143	4,5	2,166666667	2	5	5	5	4	2	5	5
Yes	Tau	68	F	4,5	960	5	0,75	2,166666667	3,428571429	5	3,4	2	2	4	4	3	3	4	2
Yes	Tau	70	F	10,5	1130	7	1,333333333	1,5	3,5	3,2			4	1	2	3	1	4	0
Yes	Tau	71	F	5,4	985	5	0,8	0,25	0,666666667	1,5	0,615384615	3	3	2	2	2	1	2	1
Yes	Tau	71	M	9,7	1315	6	0,777777778	1	1,2	1,75	0,055555556		5	5	3	3	1	3	3
Yes	Tau	74	M	7,0		6	0	1		2	4	1	0	4		2	0		
Yes	Tau	56	M	9,5		6	0	0		2,5			3	3	0	4	2	4	2
Yes	Tau	68	M	6,5		16	0	0		3	4,4	0	0	5	2	3	3	2	4
Yes	Tau	66	F	13,5		14		0			4	0	0	0	0	0	0	1	0
Yes	Tau	82	M	13,5			1	0,5		1,5			3	3	4	3	0	3	3
Yes	Tau	41	F	11,2	1230	8	0	0,571428571	2				4	4	4	4	0	3	3
Yes	Tau	74	M	7,0			0,333333333	0,75	1	5			3	4	1	4	1	2	1
Yes	Tau	75	F	13,0	915		0,5	0,833333333	0,666666667				0	3	2	4	1	1	2
Yes	Tau	66	F	6,3	895	11	0	0,666666667	0	3	3	0	1	1	3	1	0	3	1
Yes	Tau	69	F	3,0	740	11	1,625	3,5	2,25				4	5	3	5	3	3	2
Yes	Tau	73	M	9,3	1115	6	0	0,6	0,5	3			0	1	3	4	0	3	0
Yes	Tau	77	M	11,0		17	0,5	1,75	1,625	3,5	1,833333333	0	3	5	3	5	1	2	2
Yes	Tau	70	F	5,8	900	11	2,2	2,2	2,7	4,333333333	0,272727273	4	1	1	5	4	0	5	4
Yes	Tau	65	M	6,4	1035	15	0,214285714	0,666666667	0,583333333	1,4	2	1	2	3	4	3	5	5	1
