# Supplementary material for: Increase in wasteosomes (corpora amylacea) in frontotemporal lobar degeneration with specific detection of tau, TDP-43 and FUS pathology
Source: Acta Neuropathol Commun. 2024 Jun 15;12:97. doi: 10.1186/s40478-024-01812-0 (PMC11179228; doi:10.1186/s40478-024-01812-0)
Supplement: Supplementary file 2 — Supplementary Material 2. [file 40478_2024_1812_MOESM2_ESM.pdf]

Supplementary material for

INCREASE IN WASTEOSOMES (CORPORA AMYLACEA) IN FRONTOTEMPORAL LOBAR DEGENERATION WITH SPECIFIC DETECTION OF TAU, TDP-43 AND FUS PATHOLOGY

https://doi.org/10.1186/s40478-024-01812-0

Acta Neuropathologica Communications

Raquel Alsina[1,2,3], Marta Riba[1,2,3], Agnès Pérez-Millan[2,4], Sergi Borrego-Écija[4], Iban Aldecoa[5,6], Clara Romera[1,2,3], Mircea Balasa[4], Anna Antonell[4], Albert Lladó[4], Yaroslau Compta[2,3,7], Jaume del Valle[1,2,3], Raquel Sánchez-Valle[4], Carme Pelegrí[1,2,3], Laura Molina-Porcel[4,5] and Jordi Vilaplana[1,2,3]

[1]Secció de Fisiologia, Departament de Bioquímica i Fisiologia, Facultat de Farmàcia i Ciències de l'Alimentació, Universitat de Barcelona, Barcelona, Spain.
[2]Institut de Neurociències (UBNeuro), Universitat de Barcelona, Barcelona, Spain.
[3]Centros de Biomedicina en Red de Enfermedades Neurodegenerativas (CIBERNED), Madrid, Spain.
[4]Alzheimer's Disease and Other Cognitive Disorders Unit, Neurology Service, Hospital Clínic de Barcelona, Fundació de Recerca Clínic Barcelona-Institut d'Investigacions Biomèdiques August Pi i Sunyer (FRCB-IDIBAPS), Universitat de Barcelona, Barcelona, Spain.
[5]Neurological Tissue Bank of the Biobanc-Hospital Clínic-FRCB-IDIBAPS, Barcelona, Spain.
[6]Department of Pathology, Biomedical Diagnostic Center (CBD), Hospital Clínic de Barcelona, FRCB-IDIBAPS, Universitat de Barcelona, Barcelona, Spain.
[7]Parkinson's Disease and Movement Disorders Unit, Neurology Service, Hospital Clínic de Barcelona, FRCB-IDIBAPS, European Reference Network on Rare Neurological Diseases (ERN-RND), Agència de Gestió d'Ajuts Universitaris i de Recerca (AGAUR), Barcelona, Spain.

Correspondence:

Carme Pelegrí. Email: carmepelegri@ub.edu
Marta Riba. Email: mriba@ub.edu

Supplementary Figure 1 Confirmation of antibody specificity through detection of characteristic lesions of the different proteinopathies using immunofluorescence. **a** Tufted astrocyte stained with anti-p62 (green) and anti-tau (red) in the hippocampus of a PSP patient. **b** Astrocytic plaque stained with anti-p62 (green) and anti-tau (red) in the hippocampus of a CBD patient. **c** Pick body stained with (green) and anti-tau (red) in the hippocampus of a PiD patient. **d** pTDP-43 neuronal cytoplasmatic inclusion stained with anti-p62 (green) and anti-pTDP-43 (red) in the hippocampus of an FTLD-TDP patient. **e** FUS neuronal cytoplasmatic inclusion stained with anti-p62 (green) and anti-FUS (red) in the hippocampus of an FTLD-FUS patient. Nuclei are stained with Hoechst (blue). FTLD: frontotemporal lobar degeneration; PiD: Pick's disease; PSP: progressive supranuclear palsy; CBD: corticobasal degeneration. Scale bar: 10 µm.

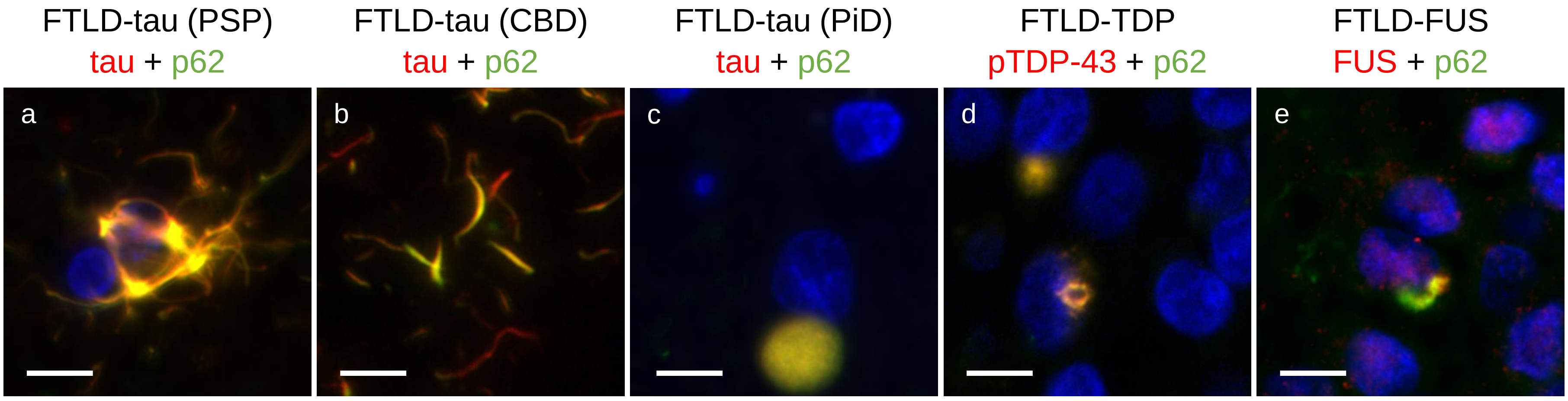

Supplementary Table 1 Primary antibodies used for immunohistochemistry and immunofluorescence.

Antibody	Clone	Company	Host
**Neuropathology**			
anti-beta-amyloid	6F/3D	Dako, Glostrup, Denmark	Mouse
anti-phospho-tau	AT8	Thermo Scientific, Rockford, USA	Mouse
anti-tau (3-repeat isoform, tau RD3)	8E6/C11	Millipore, Temecula, CA, USA	Mouse
anti-tau (4-repeat isoform, tau RD4)	1E1/A6	Millipore, Temecula, CA, USA	Mouse
anti-α-synuclein	KM51	Leica, Barcelona, Spain	Mouse
anti-α-synuclein	5G4	Roboscreen, Leipzig, Germany	Mouse
anti-phosphorylated-α-synuclein	pSyn#64	Wako Chemicals, Richmond, VA, USA	Mouse
anti-FUS	3A10B5	Proteintech, Rosemont, IL, USA	Mouse
anti-pTDP-43	11-9	Cosmo Bio, Tokyo, Japan	Mouse
anti-ubiquitin	Polyclonal	Dako, Glostrup, Denmark	Rabbit
anti-p62	3/P62 LCK ligand	BD Biosciences, San Jose, USA	Mouse
anti-α-internexin	2E3	Novex, Invitrogen, Thermo Scientific, Rockford, IL, USA	Mouse
**Immunofluorescence for protein detection in wasteosomes**			
anti-tau	Tau5	Thermo Fisher Scientific; Rockford, IL, USA	Mouse
anti-FUS	3A10B5	Proteintech, Rosemont, IL, USA	Mouse
anti-pTDP-43	11-9	Cosmo Bio, Tokyo, Japan	Mouse
anti-p62	2C11	Abcam, Cambridge, UK	Mouse

Supplementary Table 2 Description of the wasteosome score according to each area. CS: calcarine sulcus; DG: dentate gyrus; FDS: fimbriodentate sulcus; Fi: fimbria; FOV: field of view; Hc: hippocampus; HS: hippocampal sulcus; Lent: lentiform nucleus; MST: medial superior temporal; PSB: pre-subiculum; SB: subiculum; SFG: superior frontal gyrus.

<table>
<tr><th colspan="2"></th><th colspan="4">Area</th></tr>
<tr><th colspan="2"></th><th>Subpial</th><th>Periventricular</th><th>Perivascular</th><th>Intraparenchymal</th></tr>
<tr><th colspan="2">Regions</th><td>SFG, Lent, CS, MST, Hc (FDS, HS, SB, PSB)</td><td>Hc, Lent</td><td>SFG, Lent, CS, MST</td><td>SFG, Lent, CS, MST, Hc (DG, Fi)</td></tr>
<tr><th rowspan="6">Score per area</th><th>0</th><td colspan="2">No wasteosomes</td><td>No wasteosomes</td><td>No wasteosomes</td></tr>
<tr><th>1</th><td colspan="2">Isolated wasteosomes</td><td>Isolated wasteosomes in the tissue immediately after the perivascular space</td><td>1-3 wasteosomes</td></tr>
<tr><th>2</th><td colspan="2">1 row of wasteosomes</td><td>Isolate wasteosomes in the tissue immediately after the perivascular space
+ 1-3 wasteosomes in tissue close to the vessel</td><td>4-11 wasteosomes</td></tr>
<tr><th>3</th><td colspan="2">2 rows of wasteosomes (or 1 row of wasteosomes + 1-3 isolated wasteosomes close to the row)</td><td>1 row of wasteosomes in the tissue immediately after the perivascular space</td><td>12-27 wasteosomes</td></tr>
<tr><th>4</th><td colspan="2">≥ 2 rows and isolated wasteosomes close to the rows of wasteosomes</td><td>2 rows of wasteosomes in the tissue immediately after the perivascular space</td><td>28-60 wasteosomes</td></tr>
<tr><th>5</th><td colspan="2">≥ 2 rows + high density of wasteosomes surrounding the rows</td><td>≥ 2 rows of wasteosomes + high density of wasteosomes in the tissue close to the vessel</td><td>> 60 wasteosomes</td></tr>
<tr><th colspan="2">Wasteosome score</th><td>Mean value of the score given to each depth of sulci</td><td>Score given to the FOV at 400x with highest wasteosomes accumulation</td><td>Mean value of the score given to vessels of the 3 FOV at 100x</td><td>Score given to the FOV at 400x with highest accumulation of wasteosomes</td></tr>
</table>

Supplementary Table 3 Demographic and neuropathological data about the brain donors used for immunofluorescence. ARTAG: aging-related tau astrogliopathy; PMD: post-mortem delay; CBD: corticobasal degeneration; FTLD: frontotemporal lobar degeneration; FUS: Fused in Sarcoma; LATE: limbic-predominant age-related TDP-43 encephalopathy; PSP: progressive supranuclear palsy.

Subject	Sex	Age at death	PMD (hh:mm)	Neuropathological diagnosis	Copathology			
					ABC score	ARTAG in the hippocampus	LATE	FUS pathology
**1**	Female	70	11:00	FTLD-TDP (Type C)	A0B0C0	No	No	No
**2**	Male	77	07:15	FTLD-TDP (Type C)	A2B0C1	No	No	No
**3**	Male	64	10:30	FTLD-TDP (Type AB)	A1B0C0	No	No	No
**4**	Male	72	7:00	FTLD-CBD	A0B0C0	No	No	No
**5**	Female	71	04:30	FTLD-CBD	A0B0C0	No	No	No
**6**	Male	77	7:00	FTLD-PSP	A1B0C0	No	No	No
**7**	Male	78	11:00	FTLD-Pick	A1B0C1	No	No	No
**8**	Male	56	16:25	FTLD-FUS	A0B0C0	No	No	Yes
**9**	Female	54	5:47	FTLD-FUS	A1B0C0	No	No	Yes
**10**	Male	43	4:00	FTLD-FUS	A0B0C0	No	Yes	Yes
